# Supplementary material for: SyNDI: synchronous network data integration framework
Source: BMC Bioinformatics. 2018 Nov 6;19:403. doi: 10.1186/s12859-018-2426-5 (PMC6219086; doi:10.1186/s12859-018-2426-5)
Supplement: Supplementary file 5 — Files of the example presented in Additional file 4. This additional file is a zip package that contains all files used in Additional file 4. Detailed descriptions of each of these files are presented in a README file included in this zip package. (GZ 30411 kb) [file 12859_2018_2426_MOESM5_ESM.gz › SyNDITools/Meme2Fimo/meme_4.9.0_4.tar.gz!/meme_4.9.0/doc/mast2txt.html]

mast2txt


# mast2txt

**Usage:** `mast2txt <mast xml file> <output text file>`

**Description:**

Provides backwards compatibility by converting a mast xml file into a mast text file.
**Warning: Mast text format will not be supported in the next release.**

**Input:**

<mast xml file> - An xml file created by the mast program.

<output text file> - The name of the text file to output to.

**Output:**

Writes mast text output to the file name passed.
